# Targeting breast cancer with a combination of DNT and LAG3 checkpoint blockage and its mechanism

**DOI:** 10.1002/iid3.626

**Published:** 2022-07-12

**Authors:** Miao Wang, Yuhan Wei, Yingrui Li, Hongzhong Li, Jiangtao Jin, Yuting Lu, Qin Li

**Affiliations:** ^1^ Department of Oncology Beijing Friendship Hospital, Capital Medical University Beijing China; ^2^ Chongqing Key Laboratory of Molecular Oncology and Epigenetics The First Affiliated Hospital of Chongqing Medical University Chongqing China; ^3^ Department of Intervention Therapy Zezhou People's Hospital Jincheng China

**Keywords:** breast cancer, double negative T cells, immune checkpoint inhibitor, LAG3Ab, tumor immune microenvironment

## Abstract

**Introduction:**

The characteristics of the tumor immune microenvironment (TIME) are closely related to immunotherapy. Breast cancer can benefit from immunotherapy, and its TIME is still unclear.

**Methods:**

We utilized mass cytometry to explore the immune cell heterogeneity in breast cancer. Double‐negative T cells (DNTs) from healthy volunteers (HBs) were enriched in vitro. Flow cytometry was used to detect the cell surface receptors of cancer cells and DNT cells. The correlation between immune checkpoints and the abundance of immune cells or prognosis of breast cancer was analyzed by the TCGA database. The messenger RNA (mRNA) expression of functional genes was performed by quantitative real‐time PCR.

**Results:**

We found that the frequencies of Granzyme B (GZMB)^+^CD8^+^T and GZMB^+^DNT cells in cancer tissues (CA) of breast cancer were lower than those in blood samples of patients (PB), and the frequencies of programmed cell death protein 1 (PD1)^+^CD8^+^T and PD1^+^DNT cells in CA were higher than those in PB. DNTs from HBs had a cytotoxic effect on MDA‐MB‐231. LAG3Ab could upregulate the mRNA expression of interferon gamma and perforin by increasing T‐BET transcription to enhance the cytotoxicity of DNT cells in vitro.

**Conclusion:**

Our study revealed the suppressive status of TIME in breast cancer and supported DNT cells had the potential to be applied as a novel adoptive cell therapy for TNBC either alone or combined with LAG3Ab.

## Graphical abstract

We revealed the suppressive status of TIME in breast cancer and supported DNT cells had the potential to be applied as a novel adoptive cell therapy for TNBC either alone or combined with LAG3Ab.

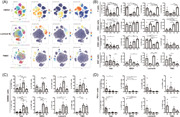

## 1 INTRODUCTION

International agency for research on cancer (https://gco.iarc.fr/) published that breast cancer has become the most common cancer among worldwide in 2020.^1^ Triple‐negative breast cancer (TNBC), accounting for 10%–20% of breast cancer, is more heterogeneous and invasive, and has the metastasis and recurrence tendencies.^2^, ^3^, ^4^, ^5^ A large proportion breast cancer patients failed to response to current treatment due to tumor immune microenvironment (TIME) heterogeneity.^6^, ^7^ The abundance and states of T cells have a great effect on tumor progression.^8^, ^9^, ^10^

Recently, immunotherapy has achieved remarkable effects in many tumors by improving host's immune response actively or passively.^7^, ^11^, ^12^ Adoptive cell therapy (ACT) has shown an encouraging efficacy in solid tumors.^11^, ^13^, ^14^ Double‐negative T (DNT) cells, defined by expressing CD3, without CD4 and CD8, has attracted more and more attention as an emerging ACT.^15^ Studies have shown that DNT cells have a significant antitumor efficacy in nonsmall cell lung cancer, pancreatic cancer, and acute myeloid leukemia.^15^, ^16^, ^17^, ^18^, ^19^, ^20^, ^21^

Immune checkpoints, such as programmed cell death protein 1 (PD1), cytotoxic T lymphocyte antigen 4, and lymphocyte activation gene 3 (LAG3), are the typical characteristic of T cell dysfunction.^22^, ^23^, ^24^ Immune checkpoint inhibitors (ICIs) have achieved great therapeutic outcomes by preventing or reversing T cell exhaustion in the TIME.^12^, ^23^, ^25^ As a promising immune checkpoint, LAG3 has attracted more and more attention. LAG3 can bind to its ligands, such as MHC II expressed on tumor cells or antigen presenting cells, to transmit inhibitory signals.^26^, ^27^ Clinical trials of LAG3 monoclonal antibody (LAG3Ab) for various cancers are being proceeded in full swing.^28^

Here we applied mass cytometry (cytometry by time of flight, CyTOF) to draw the TIME landscape of breast cancer, and depicted the frequency and distribution variation of T cells in cancer tissues (CA) and adjunct tissues (ADJ). We demonstrated the T cell cytotoxic effect on breast cancer cells in vitro, and reported the possibility of combining LAG3Ab and DNT to treat TNBC.

## 2 MATERIALS AND METHODS

### 2.1 Sample collection

CA and ADJ (2 cm away from tumor) were collected from 11 breast cancer patients who underwent surgical treatment in the General Surgery Department of Beijing Friendship Hospital, Capital Medical University. The sample volume is about 1 cm in diameter and weighs 100–200 mg. The specimen was stored in precooled RRMI‐1640 (Corning) medium and transferred to the laboratory for prompt processing immediately. Ethylenediaminetetraacetic acid tubes were used to collect 4 ml paired peripheral blood of the patients (PB) within 4 h before surgery, and 5 peripheral blood samples were obtained from healthy volunteers (HBs). This study was approved by the Ethics Committee of Beijing Friendship Hospital, Capital Medical University, and all participants signed the written informed consent.

### 2.2 Sample preparation

Tissues were processed into 1 mm^3^ fragments by surgical scissors, mixed with HBSS (Solarbio Science &Technology Co. Ltd.) containing 0.03% type IV collagenase (Sigma), 0.01% DNase I (Sigma), 10% fetal bovine serum (FBS) (Gibco), and digested by Gentle‐MACS dissociator with B‐01 mode at 37°C for 45 min. Then 10 ml Dulbecco's phosphate buffered saline (DPBS) containing 2% FBS was added to neutralize digestive enzymes. Tissues were filtered through 50‐mesh and 70‐mesh strainers, washed with DPBS containing 2% FBS, centrifuged at 300*g* for 4 min to collect precipitates. Peripheral blood mononuclear cells (PBMC) were prepared by Ficoll (DAKEWE) gradient centrifugation.

Single cells to be detected by CyTOF were stained with 0.5 μM cisplatin (Fluidigm), washed twice, fixed with 1.6% paraformaldehyde (Sigma) for 10 min at room temperature, then resuspended with cryopreserved solution containing 10% Dimethyl sulfoxide (Sigma) and cryopreserved.

Conjugated antibodies and multi‐metal labeling kits were purchased from Fluidigm. Pure‐antibodies were purchased from Biolegend and conjugated with multi‐metal labeling kits (Fluidigm) in our laboratory according to the manufacturer's instructions. The CyTOF antibody list is shown in Table S1. The cryopreserved cells were thawed and rinsed twice with cell staining buffer (CSB; Biolegend), and stained with antibodies targeting cell surface receptors at room temperature for 30 min. After incubation, the samples were processed with nuclear antigen staining perm (Fluidigm) for washing and membrane breaking and then incubated with antibodies targeting intracellular molecules at room temperature for 30 min and washed twice by DPBS. Then cells were resuspended using Ir Intercalator (Fluidigm) and stored at 4°C overnight, washed twice with CSB and Distillation‐Distillation H_2_O the next day, suspended with 10% EQ™ Four Element Calibration Beads (Fluidigm) for loading.

### 2.3 CyTOF data acquisitions and analysis

CyTOF data were obtained by a Helios™ mass cytometer (Fluidigm) from the Beijing Institute of Hepatology and analyzed visually using viSNE.^29^ viSNE is a dimensional‐reduction method implemented by Barnes‐Hut acceleration of the T‐SNE algorithm.^30^ Cytobank (http://www.cytobank.org/), FlowJo Software V10 (Treestar), and R3.6.1 (http://www.R-project.org) were applied for data analysis.

### 2.4 Cancer cell

Humanized TNBC cell line MDA‐MB‐231 and HER2+ cell line SK‐BR‐3 were purchased from China Infrastructure of Cell Line Resource. The cells were cultured with RPMI‐1640 medium containing 10% FBS in a constant temperature incubator at 37°C and 5% CO_2_. The cells were stored in a −80°C refrigerator or in liquid nitrogen using serum‐free cryopreserved solution (Cellregen).

### 2.5 Human DNT expansion

A total of 15 ml peripheral blood from HB was collected for DNT expansion. This study was approved by the Ethics Committee of Beijing Friendship Hospital, Capital Medical University (2021‐P2‐064). All participants have signed the written informed consent. PBMC was collected via Ficoll gradient centrifugation. CD4^+^ and CD8^+^ cells were removed with negative sorting kit (STEMCELL™ Technology) to enrich DNT cells from PBMC.^21^ X‐VIVO (Lonza) medium was used to purify and expand DNT cells. DNT cells were cultured in 24‐well plates coated with purified anti‐CD3 (5 μg/ml) (Biolegend) for 5 days, adding human recombinant interleukin (IL)‐2 (25 ng/ml) (peprotech) and purified anti‐CD28 (3 μg/ml) (Biolegend) at D0, D3, and D5 to the medium. Fresh X‐VIVO medium containing IL‐2 (25 ng/ml) and anti‐CD3 (100 ng/ml) was added on D7, D10 and D13. DNT cells cultured for 7–14 days were used for subsequent experiments.

### 2.6 Flow cytometry

All fluorochrome‐conjugated anti‐human antibodies against CD45 (Cat: 304062), CD3 (Cat: 300306), CD4 (Cat: 317444; Cat: 301051), CD8 (Cat: 300539), LAG3 (Cat: 369206), PD1 (Cat: 329918), NKG2D (Cat: 320808), HLA‐DR (Cat: 307604), CD274 (Cat: 329708), MICA/MICB (Cat: 320906), Annexin V (AV) (Cat: 422201), Human TruStain FcX™ (Cat: 422302) were purchased from Biolegend company. Data were collected using the Aria II Flow Cytometer (BD Biosciences), Attune NxT (Thermo), or Cytoflex (Beckman) and analyzed by FlowJo Software V10.

### 2.7 Cytotoxicity assays

DNT cells and MDA‐MB‐231 or SK‐BR‐3 cells were cocultured in 96‐well plates for 24 h with the effector to target (E:T) ratios of 0:1, 1:1, 2.5:1, 5:1, 10:1. RPMI‐1640 complete medium was used in this experiment. In some assays, DNT was prestimulated with LAG3Ab (10 μg/ml) (offered by Innovent Company) for 1 h before coculture. The specific killing of DNTs against breast cancer cells was calculated by: $\mathrm{Specific Killing}=\frac{\%{{\mathrm{CD}45}^{-}{\mathrm{Annexin V}}^{+}}_{\mathrm{with DNT}}-\%{{\mathrm{CD}45}^{-}{\mathrm{Annexin V}}^{+}}_{\mathrm{without DNT}}}{100\%-\%{{\mathrm{CD}45}^{-}{\mathrm{Annexin V}}^{+}}_{\mathrm{without DNT}}}\times 100$. In coculture systems, the apoptotic breast cancer cells were defined as CD45−Annexin V^+^.

### 2.8 Quantitative real‐time PCR

DNT cells were cultured in X‐VIVO medium with or without LAG3Ab (10 μg/ml) for 48 h, and washed twice with DPBS. Total RNA was extracted using Trizol (Sigma) reagent and complementary DNA was synthesized by Prime script™ RT Reagent Kit (Perfect Real Time) (TAKARA) according to the manufacturer's instructions. Quantitative real‐time PCR was performed by ABI 7500 Sequence Detection System (Applied Biosystems). Messenger RNA (mRNA) relative expression was calculated by $2^{-\Delta \Delta \mathrm{Ct}}$. The genes and primer sequences are shown in Table 1.

**Table 1:** Primer sequences used for quantitative real‐time PCR

Gene name	Sequence (5′→3′)
*Gapdh*	Forward: GGAGCGAGATCCCTCCAAAAT Reverse: GGCTGTTGTCATACTTCTCATGG
*Lag3*	Forward: GCCTCCGACTGGGTCATTTT Reverse: CTTTCCGCTAAGTGGTGATGG
*Klrk1*	Forward: TTTTTCAACACGATGGCAAAAGC Reverse: GGGCCACAGTAACTTTCGGT
*Perforin 1*	Forward: GACTGCCTGACTGTCGAGG Reverse: TCCCGGTAGGTTTGGTGGAA
*Granzyme b*	Forward: TACCATTGAGTTGTGCGTGGG Reverse: GCCATTGTTTCGTCCATAGGAGA
*IFNγ*	Forward: TCGGTAACTGACTTGAATGTCCA Reverse: TCGCTTCCCTGTTTTAGCTGC
*Tbx21*	Forward: GTCCAACAATGTGACCCAGAT Reverse: ACCTCAACGATATGCAGCCG

### 2.9 TCGA data analysis

TCGA data were calculated by some interactive web resources. *Lag3* expression in subgroups of breast cancer were analyzed by UALCAN (http://ualcan.path.uab.edu/).^31^ The clinical outcome relevance between *lag3* expression and cancer subtypes was determined by “outcome module” of the tumor immune estimation resource (TIMER 2.0, http://timer.cistrome.org/).^32^ Cox regression was used in this section, and the hazard ratio, *p* value for Cox model, and the log‐rank *p* value for KM curve are shown on the KM curve plot. We also applied TIMER 2.0 to explore the correlation of LAG3 (*lag3*), PD1 (*pdcd1*), NKG2D (*klrk1*) expression with immune infiltration level in breast cancer. The partial Spearman's correlation was conducted to perform this association analysis. “Gene_Corr module” of TIMER 2.0 was used to explore the correlation between interested genes in TNBC. Gene expression profiling interactive analysis (http://gepia.cancer-pku.cn/) analyzed the correlation of the pair‐wise gene expression in breast cancer. Pearson was utilized in this calculation.^33^ Relations between abundance of tumor‐infiltrating lymphocytes and *lag3* expression in diverse cancer types were evaluated by TISIDB (http://cis.hku.hk/TISIDB/).^34^

### 2.10 Statistical analysis

Experimental data was calculated by Prism 8.0 software (GraphPad Software). Comparisons were made by the Student *t* test and one‐way analysis of variance analysis. *p < .05; **p < .01; ***p < .001. Data represent SEM.

## 3 RESULTS

### 3.1 T lymphocyte cells composition in breast cancer

**Figure 1:**
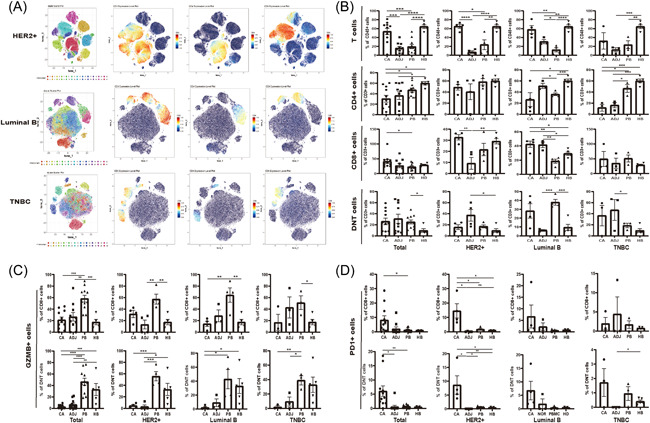
In‐depth characterization of the T cell clusters. (A) viSNE plots showing immune cell clusters and CD3, CD4, and CD8 expression in PBMCs of different breast cancer subtypes. (B) Bar plots of frequencies of T cells, CD4^+^T, CD8^+^T, and DNT cells in CA, ADJ, and PB across a total of 11 breast cancer patients, and HB of 5 healthy volunteers. (C, D) Bar plots of frequencies of GZMB^+^CD8^+^T cells (upper panel) and GZMB^+^DNT cells (lower panel) (C) and PD1^+^CD8^+^T cells (upper panel) and PD1^+^DNT cells (lower panel) (D). ADJ, adjacent tissues; CA, cancer tissues; DNT, double negative T; GZMB, Granzyme B; HB, blood samples of healthy volunteers; PB, blood samples of patients; PBMC, peripheral blood mononuclear cell; PD1, programmed cell death protein 1

To reveal the immune landscape of the TIME in breast cancer, we utilized 37 markers to analyze the immune cells in CA, ADJ, PB samples from breast cancer patients and HB samples from HBs by CyTOF. The immune cell clusters in PB from different breast cancer subtypes were shown in Figure 1A. 24 clusters were found in a HER2+ patient, 22 clusters in a Luminal B patient, and 24 clusters in a TNBC patient. The CD3, CD4, and CD8 expression variation were also found in breast cancer patients. We also observed that the frequency of T cells in CA was much higher than that in ADJ among total breast cancer patients, while the frequencies of CD4^+^T, CD8^+^T, and DNT cells in CA were comparable with those in ADJ. T cells were more enriched in CA than ADJ of HER2+ and Luminal B patients, which was not suitable for TNBC. The frequency of CD8^+^T cells in CA was higher than that in ADJ, which was only observed in HER2+ patients. Interestingly, the frequency of DNT cells in PB was higher than that in HB among total breast cancer patients and Luminal B patients (Figure 1B).

To further explore the T cells status in the TIME, we analyzed the characteristics of CD8^+^T and DNT cells. The results showed that in breast cancer population, the frequencies of Granzyme B (GZMB)^+^CD8^+^T cells and GZMB^+^DNT cells in CA were lower than those in PB, while the frequencies of PD1^+^CD8^+^T cells and PD1^+^DNT cells were higher than those in PB (Figure 1C,D).

### 3.2 Expanded DNT has a cytotoxic effect on TNBC cell in vitro

**Figure 2:**
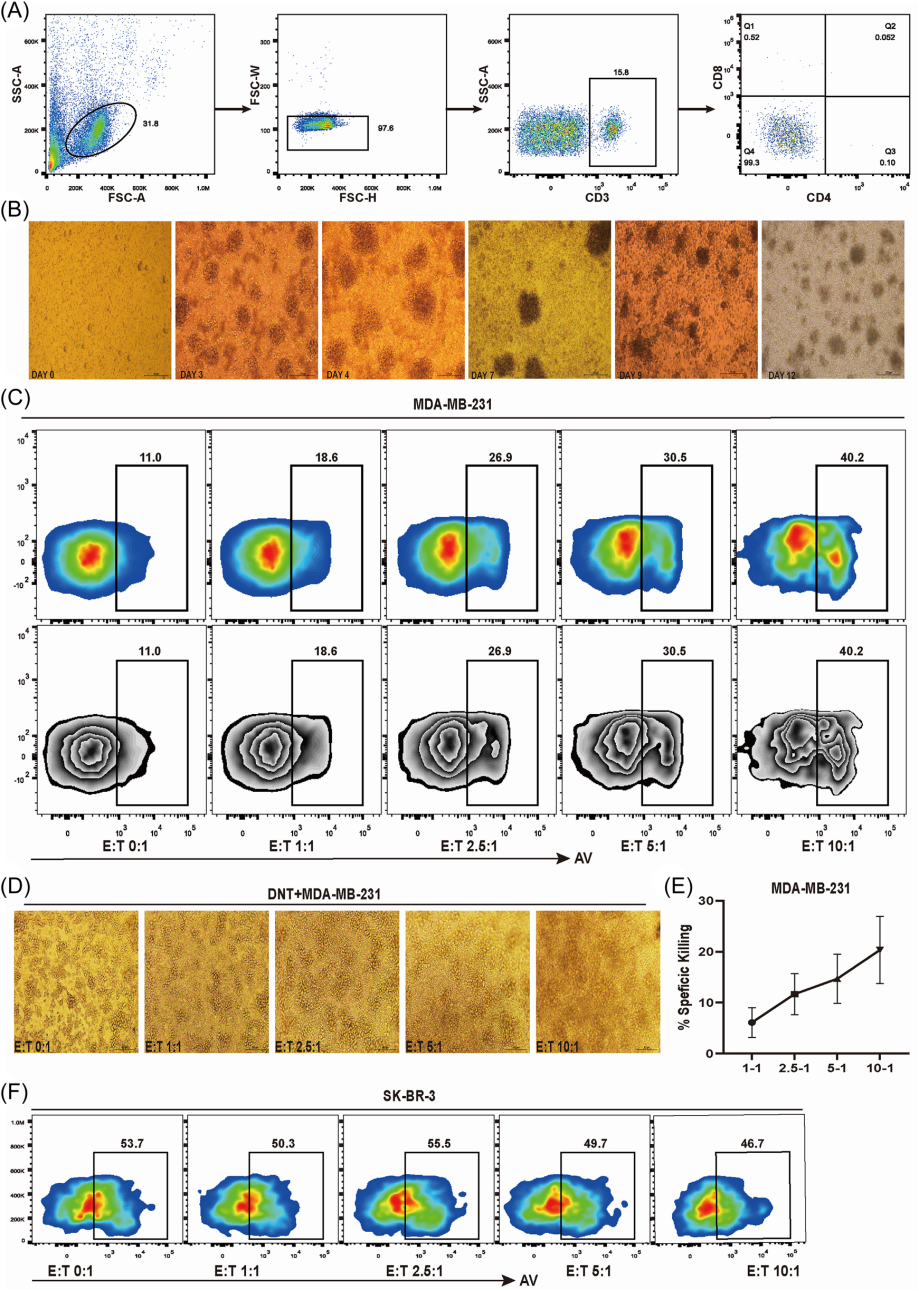
Ex vivo expanded DNTs are cytotoxic to breast cancer cells. (A) The purity of sorted DNTs. (B) The growth of DNT cells from days 0 to 12. (C–E) Expanded DNTs were cocultured with triple‐negative breast cancer cell MDA‐MB‐231 at various effector to target ratios. The cell culture status, %AV, and specific killing were shown, respectively. (F) Expanded DNTs were cocultured with HER2+ breast cancer cell SK‐BR‐3 at various effector to target ratios. %AV of tumor cells was shown. AV, Annexin V; DNT, double negative T

The purity of DNT (CD3+CD4‐CD8‐) cells obtained from HBs was above 90% (Figure 2A). DNTs expanded in vitro (Figure 2B) were cocultured with MDA‐MB‐231 or SK‐BR‐3 cells in vitro at the E:T ratios of 0:1, 1:1, 2.5:1, 5:1, and 10:1. The results showed that under different culture conditions, the apoptosis rates of MDA‐MB‐231 were 11.0%, 18.6%, 26.9%, 30.5%, and 40.2%, respectively. The specific killing increased with the upregulation of E:T ratio (Figure 2C–E). However, DNT cells barely had a cytotoxic effect on SK‐BR‐3 (Figure 2F).

### 3.3 Immune checkpoint expression on DNT

**Figure 3:**
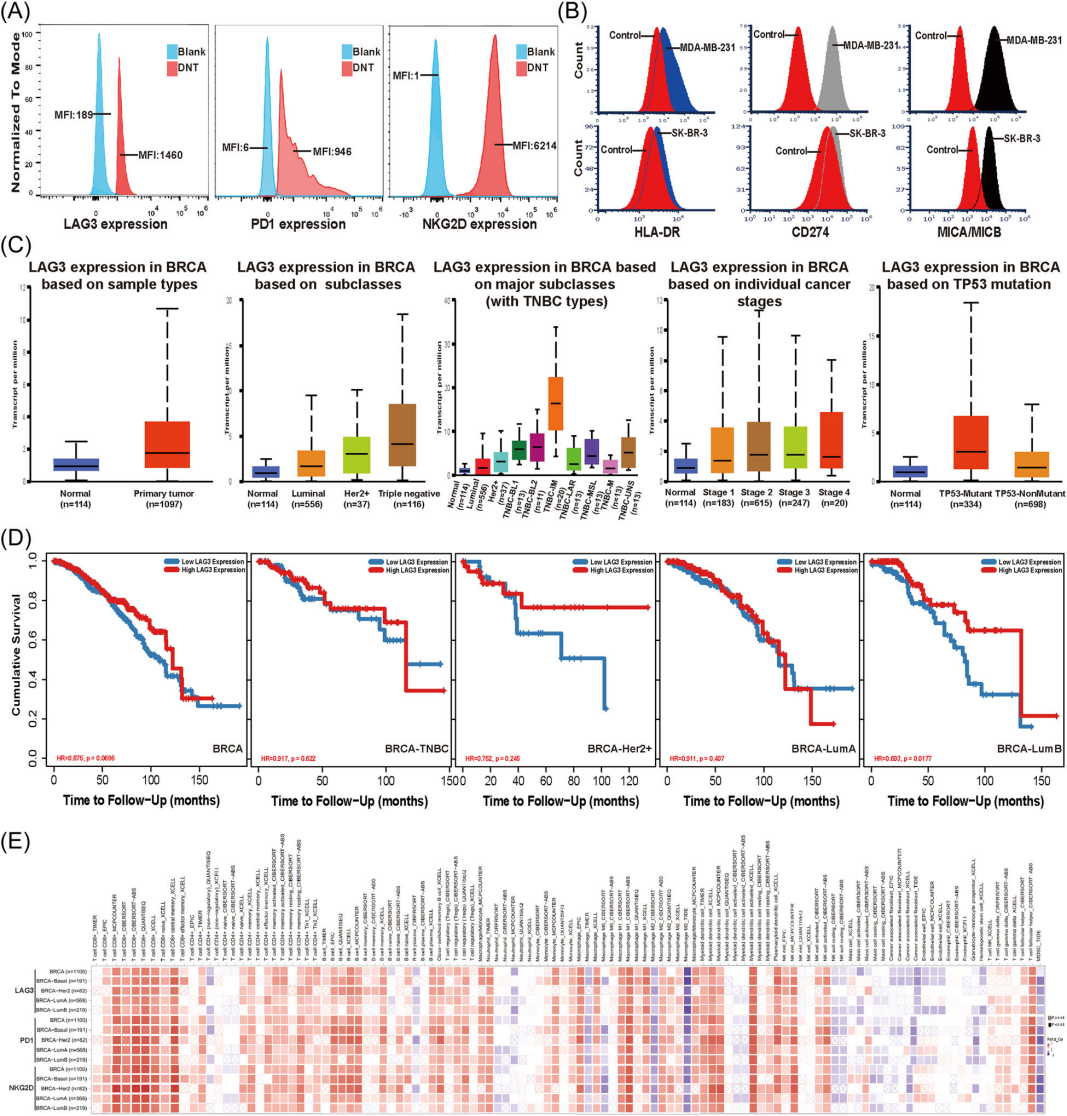
LAG3, PD1, and NKG2D expression in breast cancer from TCGA data. (A, B) Expression of LAG3, PD1, and NKG2D on DNT cells and its ligands on MDA‐MB‐231 and SK‐BR‐3 cells. Experiments were repeated twice with similar results. (C) Box‐whisker plots showing the expression of LAG3 (*lag3*) in subgroups of breast cancer samples from the TCGA database, which was tested by UALCAN. (D) Dataset analyzed by TIMER 2.0 revealed the correlation of *lag3* expression with the overall survival of different breast cancer subtype patients. (E) Spearman's correlation of LAG3 (*lag3*), PD1 (*pdcd1*), and NKG2D (*klrk1*) with immune cell infiltrations across breast cancer (TIMER). *p* < .05 was considered statistically significant. DNT, double negative T; LAG3:, lymphocyte‐associated gene 3; PD1, programmed cell death protein 1; TIMER, tumor immune estimation resource

Immune checkpoints, such as LAG3, PD1, and NKG2D, were expressed on DNT (Figure 3A), and its ligands could be detected in MDA‐MB‐231 and SK‐BR‐3 cells (Figure 3B). TCGA database analysis showed that the expression of *lag3* in breast cancer tissue was significantly higher than that in normal breast tissue, and with the highest expression in TNBC, especially in TNBC‐IM. The expression of *lag3* in TP53 mutant breast cancer was significantly higher than that in TP53 wild‐type (Figure 3C). In Luminal B, the high expression of LAG3 represents a good prognosis, while LAG3 expression was not significantly relevant to the prognosis of other subtypes of breast cancer (Figure 3D). *lag3*, *pdcd1*, and *klrk1* were positively correlated with CD8^+^T and CD4^+^T cells in breast cancer (Figure 3E).

### 3.4 LAG3Ab enhances the killing effect of DNT in vitro

**Figure 4:**
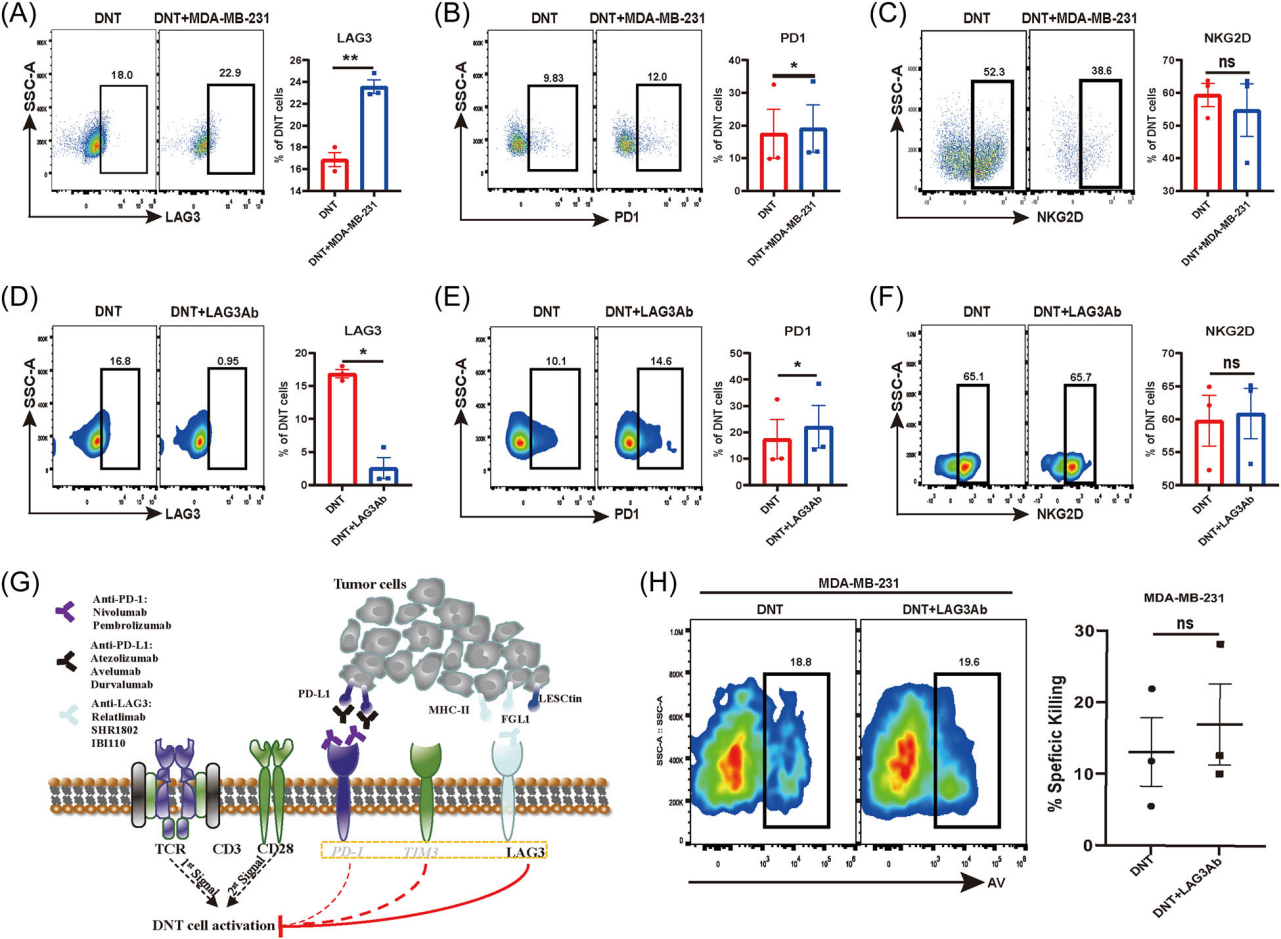
LAG3 expressed on DNTs was upregulated when cocultured with the breast cancer cell, and LAG3Ab can enhance the cytotoxic abilities of DNTs to breast cancer. (A–C) Expression of LAG3, PD1, NKG2D on DNTs when cocultured with MDA‐MB‐231. *p < .05, **p < .01, ***p < .001. The results represent three independent experiments. (D–F) Expression of LAG3, PD1, NKG2D on DNTs with or without stimulation with LAG3Ab. *p < .05, **p < .01, ***p < .001. The results represent three independent experiments. (G) LAG3Ab blocked the interaction between LAG3 on DNTs and its ligands on tumor cells. (H) LAG3Ab enhances DNT cell‐mediated cytotoxicity to the breast cancer cells. %AV and specific killing of tumor cells are shown. The results represent three independent experiments. AV, Annexin V; DNT, double negative T; LAG3, lymphocyte‐associated gene 3; LAG3Ab, LAG3 monoclonal antibody; PD1, programmed cell death protein 1

Cocultured with MDA‐MB‐231 resulted in a significant increase of LAG3^+^DNT and PD1^+^DNT cells when compared with DNT cells cultured alone (Figure 4A–C), which was consistent with the conclusion analyzed by CyTOF data that DNT cells in CA were in an exhausted state. stimulated with LAG3Ab resulted in a lower frequency of LAG3^+^DNT cells and a higher frequency of PD1^+^DNT cells (Figure 4D–F). LAG3Ab blocks the binding between LAG3 expressed on DNT cells and its ligands, such as MHC‐II, expressed on cancer cells to enhance the cytotoxicity of DNT cells (Figure 4G). In the in vitro killing assays, stimulated with LAG3Ab (10 μg/ml) could enhance the cytotoxicity of DNT cells when compared with the control group (Figure 4F).

### 3.5 LAG3Ab enhances DNT mediated antitumor activities by upregulating T‐BET

**Figure 5:**
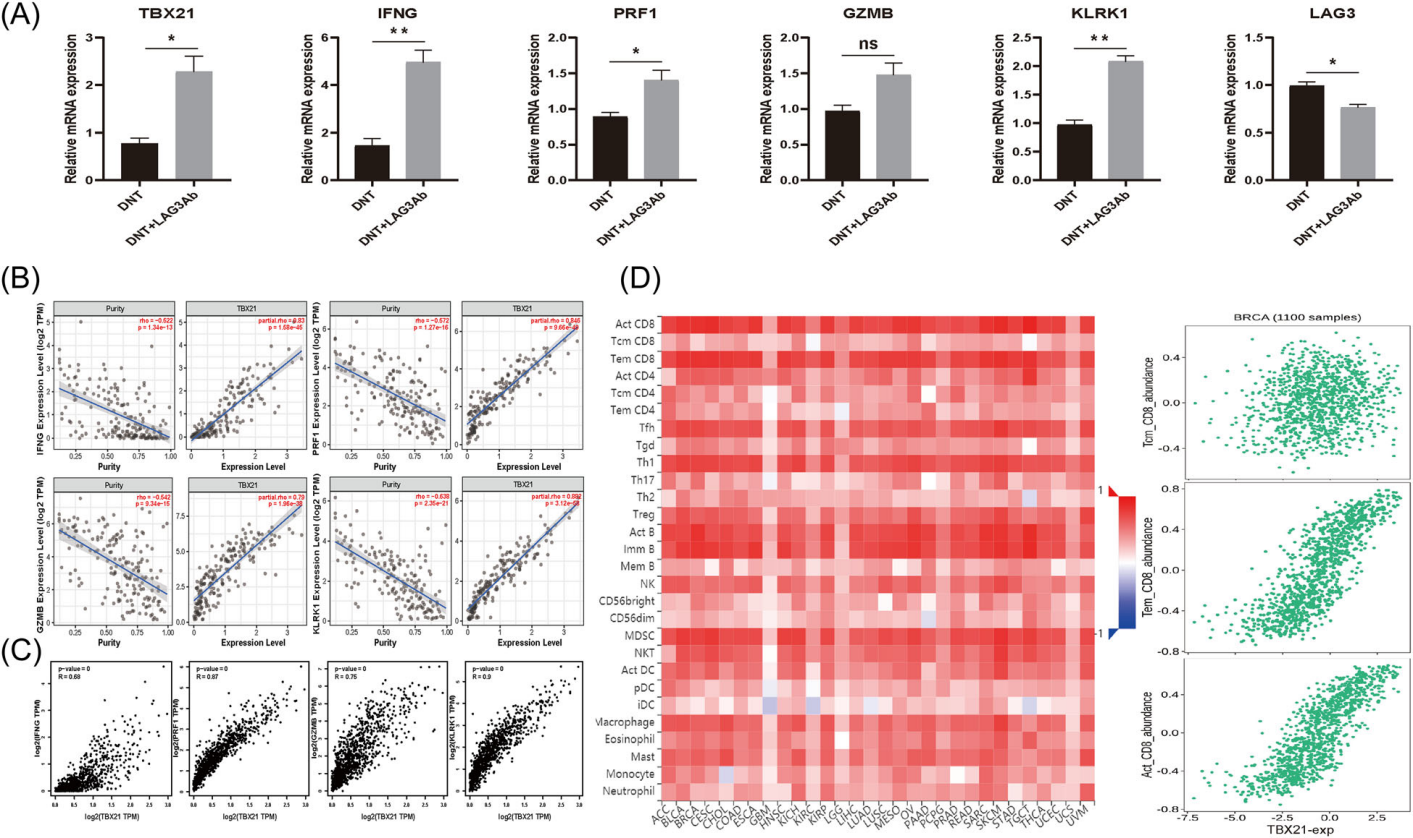
LAG3Ab increases T‐BET transcription in DNTs. The relationship between T‐BET (*tbx21)* and the activity of effector immune cells and cytotoxic cytokines. (A) Relative mRNA expression of T‐BET, IFNγ (*ifng*), Perforin (*prf1*), granzyme B (*gzmb*), NKG2D (*klrk1*), and LAG3 with or without LAG3Ab stimulation. (B, C) The correlation of T‐BET expression with indicated molecules in TNBC (TIMER) (b) and breast cancer (GEPIA) (C). (D) Dataset analyzed by TISIDB revealed the relations between *lag3* and abundance of tumor‐infiltrating lymphocytes across multi‐cancers (left) and CD8 T cell subtypes in breast cancer (right). DNT, double negative T; LAG3, lymphocyte‐associated gene 3; LAG3Ab, LAG3 monoclonal antibody; GEPIA, gene expression profiling interactive analysis; TIMER: tumor immune estimation resource; TNBC: triple‐negative breast cancer

To explore the mechanism that LAG3Ab enhances DNT cells cytotoxicity in vitro, we determined whether T‐BET (*tbx21*), a well‐known transcription factor regulating effector T‐cell activation, could be induced by LAG3Ab. Q‐PCR confirmed that LAG3Ab increased T‐BET transcription in DNT cells. IFNγ (*ifng*), perforin (*prf1*), and other cytokines transcription in DNT cells were also significantly increased after being stimulated with LAG3Ab (Figure 5A). Database mining revealed T‐BET was significantly correlative with IFNγ, perforin and other molecules in TNBC (Figure 5B) and breast cancer (Figure 5C). we also observed T‐BET was positively correlated with active CD8^+^T, Tfh and Th1 cells in multiple cancers. In breast cancer, T‐BET was more positively correlated with effector memory CD8^+^T and active CD8^+^T cells (Figure 5D).

## 4 DISCUSSION

Immunotherapy has achieved a great success in malignant solid tumors, offering an unprecedented resolution in cancer treatment.^35^, ^36^ The TIME variation affects the immunotherapy efficacy. Exploring characteristics of TIME is helpful to understand the differences of immunotherapy efficacy and select patients who benefit most from immunotherapy.^37^, ^38^ In this study, CyTOF was to reveal the proportion and distribution variation of immune cells in breast cancer, which is consistent with the different responses of breast cancer patients to immunotherapy.^39^ Although CD8^+^T and DNT cells proportion variations were observed in CA of different subtype patients, both cells are in exhausted states (Figure 1). This suggests that increasing the number of effector cells and restoring its activation in TIME is a promising way to approve the antitumor effect.

Study showed that ACT could be promising in breast cancer.^13^, ^40^, ^41^ DNT cells have achieved a remarkable efficacy in solid tumors and hematological tumors, and its cytotoxicity was comparable with CD8^+^T cells.^15^, ^16^, ^17^, ^18^, ^19^ In our study, DNT cells from HBs were purified, enriched, and expanded in vitro (Figure 2A,B). DNT would be purified again in some experiments. The frequency of MDA‐MB‐231 cell apoptosis was increased from 11.0% when cultured alone to 40.2% when cocultured with 10 times the number of DNT cells. And the average specifical killing ratio of DNT cells reached 20.34% when the E:T was 10:1 (Figure 2C–E), suggesting that TNBC patients may benefit from DNT cell therapy. While the cytotoxicity of DNT to TNBC in vivo needs further exploration. But DNT cells barely had a killing effect on SK‐BR‐3 cells, a kind of HER2+ breast cancer cell line (Figure 2F). Without the expression of CD4 and CD8 molecules, DNT cells cannot rely on the interaction of CD4 and MHC II or CD8 and MHC I to exert the antitumor effect. While according to the recent study, LAG3 expressed on DNT cells plays an important role in MHC II antigen recognition.^42^, ^43^ Our study shows that DNT cells from healthy donors expressed LAG3, and breast cancer cells expressed MHC II, which is also the main ligand of LAG3, suggesting that LAG3 makes a high contribution to the cytotoxicity of DNT cells to breast cancer. We also observed that breast cancer cells expressed a high level of MICA/MICB, which was a kind of ligands of NKG2D. DNT can depend on the connection between NKG2D receptors and its ligands to recognize cancer cells.^15^, ^16^ Expression of Granzyme B and perforin and incretion of IFNγ of DNT cells were also involved in the cytotoxicity to cancer cells,^16^, ^17^ which is a classical method to exert antitumor efficacy. The Fas/FasL pathway also plays an important role in DNT‐mediated killing effect.^16^, ^18^

Immune checkpoint is the focus of immunotherapy research, and ICIs enhance the antitumor activities of immune effector cells by blocking inhibitory pathway. Whether DNTs can be regulated by immune checkpoints and the mechanism is still unknown. Database analysis showed that LAG3 expression in breast cancer tissue was significantly higher than that in normal breast tissue, with the highest expression in TNBC‐IM; LAG3 expression in TP53 mutant breast cancer was higher than that in TP53 nonmutant breast cancer (Figure 3C). In breast cancer, *lag3*, *pdcd1*, and *klrk1* are positively correlated with the abundance of CD8^+^T and CD4+T cells (Figure 3E), suggesting that LAG3 may be a potential immunotherapeutic target in breast cancer, especially for TNBC and TP53 mutant breast cancer. Previous studies have also shown that TNBC‐IM might benefit more from ICIs,^2^ which is consistent with our conclusion. LAG3, PD1 and NKG2D molecules could be detected on DNTs (Figure 3A), suggesting DNT cells might also be regulated by immune checkpoints. We observed that stimulating DNT cells with LAG3Ab would greatly improve the cytotoxicity of DNT to breast cancer cells (Figure 4F). After prestimulation with LAG3Ab, the T‐BET transcription of DNT cells was upregulated significantly. Furthermore, the mRNA expression of cytotoxicity‐related cytokines, such as IFNγ, in DNTs were also increased after stimulation with LAG3Ab (Figure 5A). Previous studies have shown that anti‐PD1 could enhance the cytotoxic effect of T cells by upregulating the T‐BET transcription,^44^, ^45^, ^46^ which lays the foundation of co‐targeting LAG3 and PD1 in cancer immunotherapy. We demonstrated that LAG3Ab increased the cytotoxicity of DNT cells in different ways. Other studies have found that LAG3Ab may also improve the antitumor effect by positively regulating the function of CD8^+^T and CD4^+^T cells,^47^, ^48^ which provides solid evidence to target LAG3 as a novel target of immunotherapy. Studies have found that LAG3 and PD1 are co‐expressed in TNBC,^49^ and the antitumor effect of DNT cells combined with LAG3Ab and anti‐PD1 in TNBC is also worth further exploration.

In summary, we revealed that DNT and CD8^+^T cells were mostly in exhausted states in the TIME in breast cancer. DNT cells, as a novel ACT, can increase the number of immune effector cells in the TIME. DNT does not cause graft‐versus host reaction after DNT infusion, which enhances the possibility for the clinical application.^15^, ^17^ Expanded DNT cells were able to significantly kill TNBC cells in vitro in a dose dependent manner. LAG3Ab not only blocked the binding of LAG3 expressed in DNT cell surface to its ligands expressed in tumor cells, but also significantly enhanced the T‐BET transcription in DNT cells, which lays a reliable foundation for the application of ACT combined with ICIs in breast cancer, especially in TNBC.

## AUTHOR CONTRIBUTIONS

**Qin Li**: established the hypotheses, supervised the studies, and cowrote the manuscript, and had full access to all the contents included in this study and took responsibility for the integrity of the data and the accuracy of the data analysis. **Miao Wang**: participated in performing the research, analyzing the data. **Miao Wang and Hongzhong Li**: initiating the original draft of the article. **Yuhan Wei, Yingrui Li, Jiangtao Jin, and Yuting Lu**: participated in performing the research and collecting the data. All list authors participated meaningfully in the study, and they have seen and approved the submission of this manuscript.

## CONFLICTS OF INTEREST

The authors declare no conflicts of interest.

## Supporting information

Supporting information.

## Data Availability

All data generated or analyzed during this study are included.
