# Supplementary material for: Targeting breast cancer with a combination of DNT and LAG3 checkpoint blockage and its mechanism
Source: Immun Inflamm Dis. 2022 Jul 12;10(8):e626. doi: 10.1002/iid3.626 (PMC9274802; doi:10.1002/iid3.626)
Supplement: Supplementary file 1 — Supporting information. [file IID3-10-e626-s001.docx]

**Table S1**. Antibodies of mass cytometry

| Product ID | Company | Target | Clone | Label |
| --- | --- | --- | --- | --- |
| 3209003 | Fluidigm | CD11b (Mac-1) | ICRF44 | 209Bi |
| 3176010 | Fluidigm | CD4 | RPA-T4 | 176Yb |
| 3175008 | Fluidigm | CD279 (PD-1) | EH12.2H7 | 175Lu |
| 3174001 | Fluidigm | HLA-DR | L243 | 174Yb |
| 3172024 | Fluidigm | Ki-67 | B56 | 172Yb |
| 3171002 | Fluidigm | Granzyme B | GB11 | 171Yb |
| 3170001 | Fluidigm | CD3 | UCHT1 | 170Er |
| 3169003 | Fluidigm | CD25 (IL-2R) | 2A3 | 169Tm |
| 3167007 | Fluidigm | Gata3 | TWAJ | 167Er |
| 3166017 | Fluidigm | CD141 (Thrombomodulin) | M80 | 166Er |
| 3163012 | Fluidigm | BCL-6 | K112-91 | 163Dy |
| 3160010 | Fluidigm | Tbet | 4B10 | 160Gd |
| 3159028 | Fluidigm | FoxP3 | 259D/C7 | 159Tb |
| 3158018 | Fluidigm | CD324 (E-Cadherin) | DECMA-1 | 158Gd |
| 3156023 | Fluidigm | Vimentin | RV202 | 156Gd |
| 3155008 | Fluidigm | CD56 (NCAM) | B159 | 155Gd |
| 3154007 | Fluidigm | CD163 | GHI/61 | 154Sm |
| 3153020 | Fluidigm | CD185 (CXCR5) | RF8B2 | 153Eu |
| 3151020 | Fluidigm | CD278/ICOS | C398.4A | 151Eu |
| 3149011 | Fluidigm | CD127 (IL-7Ra) | A019D5 | 149Sm |
| 3148017 | Fluidigm | CD274 (PD-L1) | 29E.2A3 | 148Nd |
| 3147008 | Fluidigm | CD11c | Bu15 | 147Sm |
| 3146001 | Fluidigm | CD8a | RPA-T8 | 146Nd |
| 3145008 | Fluidigm | CD16 | 3G8 | 145Nd |
| 3143001 | Fluidigm | CD117 (ckit) | 104D2 | 143Nd |
| 3142001 | Fluidigm | CD19 | HIB19 | 142Nd |
| 3141010 | Fluidigm | HLA-ABC | W6-32 | 141Pr |
| 3089003 | Fluidigm | CD45 | HI30 | 89Y |
| 324602 | Biolegend | CD203c | / | 162Dy |
| 556059 | Biolegend | CD68 | / | 173Yb |
| 321127 | Biolegend | CD206 (MMR) | / | 144Nd |
| 555396 | Biolegend | CD14 | / | 164Dy |
| 360602 | Biolegend | CCR8 | / | 165Ho |
| 368602 | Biolegend | KLRG1 | / | 150Nd |
| 14-4877-82 | Biolegend | EOMES | / | 161Dy |
| 14-6988-82 | Biolegend | ROR gamma T | / | 168Er |
| 683402 | Biolegend | PPARr | / | 152Sm |
